# Prospective associations between parental warmth and knowledge in late adolescence and depression and anxiety symptoms in young adulthood: a Swedish cohort study

**DOI:** 10.1186/s12889-026-27712-7

**Published:** 2026-05-14

**Authors:** Maria Granvik Saminathen, Andreas Lundin, Jonas Raninen, Sara Brolin Låftman

**Affiliations:** 1 Department of Public Health Sciences, Stockholm University, Stockholm, SE-106 91 Sweden; 2 Department of Global Public Health, Karolinska Institutet, Stockholm, SE‑171 77 Sweden; 3 Centre for Epidemiology and Community Medicine, Region Stockholm, Box 45436, Stockholm, SE‑104 31 Sweden; 4 Department of Clinical Neuroscience, Karolinska Institutet, Stockholm, SE‑171 77 Sweden; 5 Centre for Alcohol Policy Research, La Trobe University, Melbourne, Australia

**Keywords:** Adolescents, Parents, Support, Knowledge, Monitoring, Depression, Anxiety, Longitudinal

## Abstract

**Background:**

Parenting practices, such as emotional support and warmth, are key determinants of adolescent mental health, whereas the role of parental knowledge is less well understood. Parental warmth is thought to foster openness and disclosure, raising the question of whether parental knowledge independently predicts mental health outcomes or primarily reflects the quality of the parent-adolescent relationship. The present study used prospective data to test whether parental knowledge in late adolescence contributes independently to depression and anxiety symptoms in young adulthood after accounting for parental warmth and relevant covariates.

**Methods:**

Data from two waves of the national Swedish cohort study Futura01 were used (*n* = 2,697). Parental warmth and parental knowledge were measured at age 18, each using two items. Depression and anxiety symptoms were measured at age 21 with the Patient Health Questionnaire-4 (PHQ-4). Covariates included participant sex, parental education, immigration background, living arrangements at age 18, and indicators of mental health problems at age 18. Linear probability models were performed.

**Results:**

In unadjusted analyses, higher parental warmth and parental knowledge during late adolescence were both associated with a lower probability of reporting depressive and anxiety symptoms in young adulthood. However, when mutually adjusting for parental warmth and parental knowledge, the effect of parental knowledge was no longer statistically significant, indicating that its association with mental health outcomes was confounded by parental warmth. In contrast, parental warmth remained significantly associated with lower probabilities of both depression and anxiety symptoms, even after accounting for parental knowledge, sociodemographic characteristics, and earlier mental health problems. There were no significant interactions between parental warmth and parental knowledge, and participant sex did not moderate the associations.

**Conclusion:**

This study highlights the protective role of parental warmth during late adolescence for depression and anxiety symptoms in young adulthood, with warmth showing a consistent association with lower probabilities of these outcomes. However, parental knowledge did not demonstrate an independent effect on mental health after accounting for warmth and covariates. The findings underscore the importance of fostering warm, supportive parent–adolescent relationships during late adolescence, as these relationships appear to have lasting benefits into young adulthood.

**Supplementary Information:**

The online version contains supplementary material available at 10.1186/s12889-026-27712-7.

## Background

Globally, both adolescents and young adults have been found to be disproportionally affected by mental health disorders such as depression and anxiety [1], particularly young women [2]. Such disorders have a typical first onset before age 25 [3], making early prevention during childhood and adolescence key for improving long-term mental health outcomes. Over recent decades, high-income countries have seen a significant rise in self-reported psychological distress among adolescents and young adults [4]. In Sweden, this trend is particularly pronounced, with poor mental health among young people emerging as a growing public health problem. Internalizing problems such as depressive and anxiety symptoms have become increasingly prevalent [5], underscoring the urgent need for effective strategies and interventions to support the mental well-being of young people [6].

Identifying modifiable protective factors is central for enhancing mental well-being among adolescents and young adults. Parental knowledge, defined as parents’ awareness of their children’s whereabouts and activities, is one such factor that has been linked to mental health outcomes. However, the link between parental knowledge and young people’s mental health has been called into question [7], as the process through which parents obtain information about their children’s whereabouts and activities during adolescence are likely to be strongly linked to the quality of the parent-adolescent relationship. Thus, factors such as parental warmth and support may confound the association between parental knowledge and adolescent internalizing symptoms, as adolescents are more likely to disclose information when parents are emotionally supportive [8, 9].

Parental warmth, commonly characterized by verbal and non-verbal expressions of love, enthusiasm, and positive regard, has been consistently linked to lower depression rates in adolescents and young adults. These behaviors convey safety, acceptance, and attentiveness to the child or adolescent’s needs and have been shown to be essential to the individual’s socioemotional development [10]. Several reviews and meta-analyses have consistently identified parental warmth as a key protective factor against internalizing symptoms in adolescents. Findings show that higher parental warmth is linked to reductions in depression and anxiety, while low warmth increases the risk for these mental health issues over time [11–13].

### Parental knowledge and mental health

However, the role of parental knowledge in young people’s mental health is less clear. High levels of parental knowledge are often attributed to effective parental monitoring. Despite the complexities surrounding the overarching concept of ‘parental monitoring’, research has established a notable link between various aspects of parental monitoring and mental health outcomes in young people. A meta-analysis by Yap et al. [11] suggested a small but significant association between increased parental monitoring and reduced risk for depression among adolescents between 12 and 18 years. However, the evidence for a connection between parental monitoring and anxiety is mixed. In Yap et al.’s meta-analysis, one study linked higher parental monitoring to fewer anxiety symptoms, while another found it predicted greater anxiety. Similarly, a prospective study by Cadman et al. [14] found that higher levels of parental monitoring in early adolescence, encompassing solicitation, parental control, adolescent disclosure, and parental knowledge, was associated with a reduced risk of major depressive disorder and anxiety disorder in late adolescence. The study by Cadman et al. accounted for the emotional quality of the parent-child relationship, but since their measure of parental monitoring incorporated both parental knowledge and child disclosure, the authors suggest that this operationalization of monitoring may, in part, capture open and trusting child-parent relationships, which could in turn be conducive for adolescent mental health [14].

The mechanisms through which parental knowledge could reduce depression and anxiety symptoms in adolescents remain unclear [7]. There are two potential pathways. The first pathway is that parental knowledge primarily reflects the overall quality of the parent-adolescent relationship. In this pathway, parental warmth may act as a confounder, assuming that it shapes both parental knowledge and mental health outcomes. Adolescents who feel cared for and listened to may be more likely to disclose information, fostering a supportive environment that protects against internalizing symptoms [15]. Warm and caring parent-child relationships may enhance parental knowledge, as adolescents are inclined to spend more time with their parents and share details of their lives, including about their peers and whereabouts [16]. This idea is supported by Liu et al. [17], who found in a meta-analysis that adolescents are more likely to disclose personal information when they perceive their parents as warm and supportive. The second pathway is that parental knowledge may independently benefit mental health by providing opportunities for guidance and support. Parents who are well-informed about their child’s challenges may be better positioned to offer timely assistance and facilitate access to professional help when needed [8, 14].

### Supportive parenting during the transition into young adulthood

Late adolescence (18–19 years) is a key period that sets the foundation for future development, as individuals begin making choices and engaging in activities that direct the course of their lives [18].

While adolescence marks a transition away from the family of origin and towards independence, parents remain a key source of guidance, support, and stability, continuing to shape emotional and psychological development into young adulthood [19]. Yet, this phase is often accompanied by shifting dynamics in the parent-child relationship, including an increase in conflict and a potential decrease in warm interactions [20]. These changes can negatively impact young people’s mental well-being [12], whereas positive parenting throughout the full span of adolescence has been linked to better mental health outcomes both in the present and later in life [21–23].

### The present study

Building on prior research, the current study examines how parental warmth and knowledge during late adolescence predict depression and anxiety symptoms in young adulthood. While parental warmth is widely recognized as a protective factor for young people’s mental health, its relationship with parental knowledge introduces nuance. Emotional support may foster adolescent disclosure, which in turn increases parental knowledge about their child’s whereabouts and activities. However, high levels of parental knowledge do not necessarily stem solely from warm relationships. They may also reflect active monitoring efforts, which can occur independently of emotional warmth.

Further, parental warmth alone does not guarantee high parental knowledge. Parents may demonstrate emotional support while remaining less involved in monitoring their adolescent’s activities, resulting in lower overall parental knowledge than would be observed with greater monitoring efforts, even if the adolescent is more likely to be open about their lives. As a result, parents may be less responsive to potential mental health problems or other situations that require an intervention. This duality underscores the need to disentangle the independent contributions of parental warmth and knowledge to mental health outcomes in young adulthood.

Unlike previous studies that have primarily examined parental knowledge or focused on earlier developmental stages, this study uses prospective data to test whether parental knowledge contributes independently to depression and anxiety symptoms in young adulthood after accounting for parental warmth and relevant covariates. By focusing on late adolescence, a critical yet understudied transition period, we aim to clarify the unique role of parental knowledge in shaping mental well-being during the transition to adulthood.

Prior research shows that parent-child relationships can vary according to sociodemographic characteristics, such as sex [24], parental education [25], immigration background [26], and living arrangements [27]. These characteristics have also been shown to be associated with depression and anxiety [28], making them important covariates in analyses of parental warmth and mental health. In addition, to clarify the temporal ordering between parental warmth and later mental health outcomes, it is essential to adjust for individuals’ prior mental health status.

Thus, by examining whether parental knowledge has an independent effect after accounting for parental warmth and relevant covariates, this study aims to clarify the unique role each factor plays in shaping mental well-being during the transition to adulthood. Based on the literature reviewed, we hypothesize the following:

1. Higher levels of parental warmth during late adolescence will be associated with lower levels of depression and anxiety symptoms in young adulthood.
2. Higher levels of parental knowledge during late adolescence will be associated with lower levels of depression and anxiety symptoms in young adulthood.
3. The association between parental knowledge and mental health outcomes will be attenuated when controlling for parental warmth, indicating that parental warmth may partly confound this relationship.

## Methods

### Data and participants

The data for this study was drawn from Futura01, a nationally based prospective cohort of individuals born in 2001. The first wave of data was collected in 2017 when participants attended grade 9 of compulsory school, age 15 or 16 years, depending on what month they were born (*n* = 5,537). A random sample of 500 schools (and one class per school) across Sweden were selected, and 343 schools (69%) participated in the first wave of the survey. In these schools, 5,722 students (85%) agreed to participate and completed a class room questionnaire. The participating and non-participating schools did not differ significantly in terms of average academic merit scores for grade nine, the proportion of parents with higher education, or the proportion of parents with an immigration background [29]. After excluding individuals due to reasons such as incomplete personal identity numbers, the final baseline sample consisted of 5,537 participants. The second wave was offered as a web survey or postal survey to the participants, who had now reached the age of 17 or 18 years, with 4,141 participants responding (75%). In the third wave that took place as a web survey when participants were 20 or 21 years old, 2,956 of those who participated in both waves 1 and 2 responded (71% of respondents in wave 2). Data from administrative registers have been linked to the survey data. This study was based on survey data from waves 2 and 3 as well as register information. The study sample included 2,697 individuals with complete values on all variables included in this study, i.e., 91% (2,697/2,956 = 0.91) of all of those who participated in all three waves).

### Measures

#### Depression and anxiety symptoms

Were measured at age 21 with the 4-item Patient Health Questionnaire-4 (PHQ-4), a brief self-report instrument that includes the 2-item depression scale Patient Health Questionnaire-2 (PHQ-2) and the 2-item anxiety scale Generalized Anxiety Disorder-2 (GAD-2) [28]. The PHQ-4 consists of the following question: “Over the last two weeks, how often have you been bothered by the following problems?”. Depression symptoms are measured through the items “feeling down, depressed or hopeless” and “little interest or pleasure in doing things”. The items capturing anxiety symptoms are “feeling anxious, nervous or on edge” and “not being able to stop or control worrying”. Each item had the response categories “Not at all” (0); “Several days” (1); “More than half the days” (2); and “Nearly every day” (3). Scores for each item pair were summed together to a scale ranging 0–6. For both PHQ-2 and GAD-2, 3 was chosen as cutoff point, creating binary variables for depression and anxiety symptoms, respectively. These cutoffs have been suggested as thresholds for identifying individuals at risk for major depressive disorder and generalized anxiety disorder [30]. However, we also performed the analyses using the full scales (0–6), with results presented in the Supplementary Material.

#### Parental warmth and parental knowledge

Were measured at age 18 through two items each, with response alternatives ranging from 1 (almost never) to 5 (almost always). Parental warmth was measured using the statements: “I can easily get affection and care from my mother and/or father” and “I can easily get emotional support from my mother and/or father”. Parental knowledge was assessed using the statements: “My parent(s) know(s) whom I spend time with during evenings” and “My parent(s) know(s) where I am during evenings”. Mean values were calculated for both parental warmth and parental knowledge by averaging the two items for each construct (i.e., summing the item scores and dividing by two), resulting in variables that retained the original 1–5 response scale. The correlation between the two measures was moderate (*r* = 0.38, *p* < 0.001) [31].

#### Sex

Was determined based on information from participants’ personal identity number, categorizing individuals as either “female” or “male”. While the register data only provided binary information on sex, it is important to recognize that sex and gender are intertwined, with gender reflecting socially constructed roles and identities that may influence both predictors and outcomes. Given these complexities, the measure used in this study reflects aspects of both sex and gender, acknowledging their interplay in shaping mental health outcomes.

#### Living arrangements

Was measured at age 18 by the question “How do you live?” with the response categories “live with mother and father”; “live with mother”; “live with father”; “live about half of the time with mother and half of the time with father (shared residence)”, “lives in own accommodation” and “other”. Five categories were formed: “two original parents”, “single parent”, “shared residence”, “independent housing”, and “other or missing”.

Information on *parental education* was obtained from register data on father’s and mother’s educational level at baseline in 2017. A variable indicating the highest parental educational level in three categories was created: (1) two years upper secondary school or less; (2) ≥ three years upper secondary school; and (3) tertiary education.

#### Immigration background

Was based on register information about the country of birth of the participants and the participants’ parents. A variable was constructed distinguishing between participants (1) born in Sweden with at least one parent born in Sweden; (2) born in Sweden with both parents born abroad; and (3) born abroad.

Given that psychosomatic complaints in adolescence can indicate underlying depression and anxiety symptoms and are linked to mental health outcomes in young adulthood [32–34], analyses were further adjusted for *psychosomatic complaints* at age 18. As the PHQ-4 instrument was only introduced in the third wave of the survey (age 21), these complaints serve as a proxy for prior mental health, aiming to account for pre-existing symptoms of depression and anxiety. Psychosomatic complaints were assessed using the survey question: “During the past six months, how often have you had …” followed by the items “stomach ache”, “headache”, and “difficulties falling asleep”. Response categories for all items were: “every day,” “a few times a week,” “once a week,” “sometime a month,” and “less often or never”. Responses were combined into an index ranging from 3 to 15 [31, 35].

To further account for prior mental health problems, analyses controlled for *the use of medication for depression* and *anxiety* at age 18. Participants were asked whether they had ever been prescribed such medication, with response options of “yes” or “no”.

Adjusting for psychosomatic complaints and medication use allows for a more accurate estimation of the impact of parenting practices on later mental health by accounting for earlier mental health symptoms.

### Statistical analysis

Descriptive statistics were presented to summarize the data. To assess the prospective associations between parental warmth and parental knowledge in late adolescence and depression and anxiety symptoms in young adulthood, we performed linear probability models (LPM), presenting unstandardized regression coefficients (b) with 95% confidence intervals (CI). Linear probability models were used because the coefficients can be interpreted as changes in probability, offering a clearer interpretation than odds ratios from logistic regressions, while also allowing for comparisons across models [36]. Model 1 included only parental warmth, while Model 2 included only parental knowledge. Model 3 mutually adjusted for parental warmth and knowledge, while Model 4 further adjusted for sociodemographic characteristics and indicators of earlier mental health problems. To account for clustering at the school level, robust standard errors clustered by school at baseline were used. The number of schools was 335. Interaction analyses were conducted to test for departure from additivity by including interaction terms in the fully adjusted models, examining whether parental warmth and parental knowledge interacted in predicting depressive and anxiety symptoms and whether these associations varied by sex. Multicollinearity was assessed using variance inflation factors (VIF), and all predictors showed VIF values well below the conventional threshold of 10, indicating no concerns regarding multicollinearity. Stata was used for all analyses [37].

### Ethics

The study received ethical approval from the Swedish Ethical Review Authority (ref. 2021-06504-01; 2022-02781-02; 2022-06502-02), and all participants provided written informed consent prior to their involvement.

#### Large language models (LLMs)

ChatGPT was used to check the grammar, proofread the text and provide clarifications.

## Results

Descriptive statistics for all study variables are presented in Table 1. Descriptives of the variables among all individuals who participated in all three waves is provided in the Supplementary Material, Table S1. A correlation matrix of the continuous variables is provided in the Supplementary Material, Table S2.

**Table 1:** Descriptive statistics of the study sample (*n* = 2,697)

	*n*	%
Individual and parental background variables		
Sex		
Males	1,132	42.0
Females	1,565	58.0
Parental education		
Tertiary	1,802	66.8
≥ 3 years secondary	509	18.9
≤ 2 years secondary	386	14.3
Immigration background		
Born in Sweden, at least one parent born in Sweden	2,257	83.7
Born in Sweden, both parents born abroad	207	7.7
Born abroad	233	8.6
Age 18		
	n	%
Living arrangements		
Two original parents	1,744	64.7
Single parent	404	15.0
Shared residence	305	11.3
Lives in own accommodation	60	2.2
Other/missing	184	6.8
Medication depression	123	4.6
Medication anxiety	136	5.0
	Mean	s.d.
Parental warmth	4.3	1.0
Parental knowledge	4.5	0.8
Psychosomatic complaints	7.2	2.7
Age 21		
	n	%
Depressive symptoms	678	25.1
Anxiety symptoms	724	26.8

Table 2 shows the results of linear probability models examining the associations between parental warmth and knowledge at age 18 and depression symptoms at age 21. Model 1 shows that higher levels of parental warmth at age 18 were associated with a lower probability of depression symptoms at age 21 (b = -0.06, 95% CI -0.08, -0.04). Model 2 shows that higher levels of parental knowledge at age 18 were linked to a lower probability of depression symptoms at age 21 in the unadjusted analysis (b = -0.04, 95% CI -0.06, -0.02). When mutually adjusting for parental warmth and knowledge in Model 3, the coefficient for parental warmth remained unchanged (b = -0.06, 95% CI -0.08, -0.04). By contrast, the coefficient for parental knowledge was attenuated and no longer statistically significant (b = -0.02, 95% CI -0.04, 0.01, *p* = 0.177). In Model 4, which added sociodemographic characteristics and indicators of earlier mental health problem, the coefficient of parental warmth remained statistically significant (b = -0.04, 95% CI -0.06, -0.02), while the effect of parental knowledge remained non-significant (b = -0.01, 95% CI -0.03, 0.01, *p* = 0.266). We tested interactions between parental warmth and parental knowledge, as well as between sex and each parenting practice. However, none of these interactions were statistically significant (not presented in Table). Analyses of the continuous PHQ-2 measure (scale 0–6) is provided in the Supplementary Material, Table S3. These analyses show patterns similar to those observed with the dichotomous measure.

**Table 2:** Results from linear probability models of depressive symptoms at age 21, by parenting practices at age 18. Coefficients and 95% confidence intervals (CI) (*n* = 2,697)

	Model 1^a^		Model 2^b^		Model 3^c^		Model 4^d^	
	b	95% CI	b	95% CI	b	95% CI	b	95% CI
Parental warmth (age 18)	-0.06***	-0.08, -0.04			-0.06***	-0.08, -0.04	-0.04***	-0.06, -0.02
Parental knowledge (age 18)			-0.04***	-0.06, -0.02	-0.02	-0.04, 0.01	-0.01	-0.03, 0.01
Sex								
Males (ref.)							0.00	
Females							-0.01	-0.05, 0.02
Parental education								
Tertiary (ref.)							0.00	
≥ 3 years secondary							0.01	-0.03, 0.05
≤ 2 years secondary							0.04	-0.01, 0.10
Immigration background								
Born in Sweden, at least one parent born in Sweden (ref.)							0.00	
Born in Sweden, both parents born abroad							0.12**	0.06, 0.19
Born abroad							0.07*	0.02, 0.13
Living arrangements (age 18)								
Two original parents (ref.)							0.00	
Single parent							0.01	-0.04, 0.06
Shared residence							0.00	-0.05, 0.04
Lives in own accommodation							-0.05	-0.14, 0.05
Other/missing							0.04	-0.03, 0.11
Psychosomatic complaints (age 18)							0.02***	0.02, 0.03
Medication depression (age 18)							0.16*	0.04, 0.29
Medication anxiety (age 18)							0.04	-0.08, 0.16
R^2^	0.02		0.01		0.02		0.07	

***p < 0.001 **p < 0.01 *p < 0.05

^a^ Includes only parental warmth

^b^ Includes only parental knowledge

^c^ Mutually adjusts for parental warmth and parental knowledge

^d^ Mutually adjusts for parental warmth, parental knowledge, and covariates

Table 3 presents the results of linear probability models assessing the links between parental warmth and knowledge at age 18 and anxiety symptoms at age 21. Model 1 shows that higher levels of parental warmth at age 18 were associated with a lower probability of anxiety symptoms at age 21 in the unadjusted analysis (b = -0.08, 95% CI -0.09, -0.06). Model 2 shows that higher levels of parental knowledge at age 18 were associated with a slightly lower probability of reporting anxiety symptoms at age 21 (b = -0.02, 95% CI -0.04, 0.00, *p* = 0.041). In Model 3, the effect of parental warmth remained unchanged (b = -0.08, 95% CI -0.10, -0.06) whereas the association for parental knowledge was non-significant (b = 0.02, 95% CI -0.01, 0.04, *p* = 0.150). In Model 4, which included both parental warmth and knowledge as well as sociodemographic characteristics and earlier indicators of mental health problems, the coefficient for parental warmth remained statistically significant (b = -0.06, 95% CI -0.08, -0.04), whereas the coefficient for parental knowledge remained non-significant (b = 0.01, 95% CI -0.01, 0.03, *p* = 0.478). Interactions between parental warmth and parental knowledge, and between sex and each parenting practice, were tested, but none reached statistical significance (not presented in Table). Analyses of the continuous GAD-2 measure (scale 0–6) is provided in the Supplementary Material, Table S4. The patterns remain consistent with those obtained using the dichotomous measure.

**Table 3:** Results from linear probability models of anxiety symptoms at age 21, by parenting practices at age 18. Coefficients and 95% confidence intervals (CI) (*n* = 2,697)

	Model 1^a^		Model 2^b^		Model 3^c^		Model 4^d^	
	b	95% CI	b	95% CI	b	95% CI	b	95% CI
Parental warmth (age 18)	-0.08***	-0.09, -0.06			-0.08***	-0.10, -0.06	-0.06***	-0.08, -0.04
Parental knowledge (age 18)			-0.02*	-0.04, 0.00	0.02	-0.01, 0.04	0.01	-0.01, 0.03
Sex								
Males (ref.)							0.00	
Females							0.11***	0.08, 0.15
Parental education								
Tertiary (ref.)							0.00	
≥ 3 years secondary							-0.01	-0.05, 0.03
≤ 2 years secondary							-0.01	-0.06, 0.04
Immigration background								
Born in Sweden, at least one parent born in Sweden (ref.)							0.00	
Born in Sweden, both parents born abroad							0.05	-0.02, 0.12
Born abroad							0.02	-0.04, 0.07
Living arrangements (age 18)								
Two original parents (ref.)							0.00	
Single parent							0.01	-0.03, 0.06
Shared residence							0.02	-0.04, 0.07
Lives in own accommodation							-0.01	-0.13, 0.11
Other/missing							0.03	-0.03, 0.10
Psychosomatic complaints (age 18)							0.03***	0.02, 0.04
Medication depression (age 18)							0.07	-0.04, 0.19
Medication anxiety (age 18)							0.12*	0.01, 0.24
R^2^	0.03		0.00		0.03		0.11	

***p < 0.001 **p < 0.01 *p < 0.05

^a^ Includes only parental warmth

^b^ Includes only parental knowledge

^c^ Mutually adjusts for parental warmth and parental knowledge

^d^Mutually adjusts for parental warmth, parental knowledge, and covariates

## Discussion

The present study examined the associations between parental warmth and parental knowledge during late adolescence and mental health outcomes in young adulthood within a cohort of young people in Sweden. Based on prior research, we hypothesized that higher levels of parental warmth and knowledge would be associated with lower levels of depression and anxiety symptoms in young adulthood, and that the association between parental knowledge and the mental health outcomes would be attenuated when controlling for parental warmth. Our findings showed that higher parental warmth at age 18 was consistently associated with a lower probability of reporting depression and anxiety symptoms at age 21, even after adjusting for sociodemographic characteristics and earlier mental health problems. However, parental knowledge was not significantly associated with either depression or anxiety symptoms when mutually adjusting for parental warmth (Tables 2 and 3). This lack of independent effect raises important questions about the role of parental knowledge in late adolescence, and warrants further exploration.

Our findings align with prior research highlighting the protective role of parental warmth for young people’s mental health during the transition to adulthood [12, 13], supporting the idea that caring interactions with parents foster security and emotional stability, promoting mental well-being. The results further show that parental warmth during adolescence is linked to lower levels of internalizing symptoms beyond adolescence, which is in line with findings by Ford et al. [22], who observed that adolescents who reported warm, loving, close, and caring relationships with their parents experienced better long-term mental health outcomes in the third decade of life. This suggests that a supportive relationship with parents during adolescence, even once adolescents are of legal age, continues to benefit mental health as young people navigate the challenges of young adulthood. However, research by Fang et al. [38] found no significant link between parental warmth in adolescence and depression at ages 23 or 25, suggesting that the effects of positive parenting practices may not persist throughout the young adulthood years.

As reviewed above, studies have linked parental monitoring and, more specifically, parental knowledge with improved mental health outcomes among adolescents [11, 14]. However, parental knowledge was not independently associated with depression or anxiety symptoms in this study. This suggests that the apparent association between parental knowledge and mental health in unadjusted analyses may reflect confounding by parental warmth, rather than a direct effect of knowledge itself. Our study did not test indirect pathways or mechanisms, so any potential role of parental knowledge in facilitating guidance or support should be explored in future research.

Several alternative explanations for this null finding warrant consideration. Firstly, the null result may reflect measurement limitations. The available measure focused exclusively on parents’ awareness of their adolescents’ whereabouts during evenings, which may not fully capture the broader construct of parental knowledge that encompasses a wider range of contexts, including daytime activities, digital peer interactions, and school-related experiences. The restricted focus on evenings could underestimate the true extent of parental knowledge and its potential associations with mental health outcomes. Additionally, the brevity of the scale (two items) and potential ceiling effects may have limited our ability to detect meaningful variation in parental knowledge. Future studies could employ more comprehensive measures to better assess the role of parental knowledge.

Another possibility is that parental knowledge may indeed matter less for future mental health outcomes during adolescence. During early and middle adolescence, parents’ awareness of their children’s activities and peer relationships may help prevent risky behaviors and support emotional well-being [8, 14]. Young people’s growing independence by late adolescence may reduce the impact of parental knowledge, and the transition to young adulthood might also shift the focus of parent-adolescent relationships from monitoring to emotional support and warmth.

An intriguing pattern emerged in the association between parental knowledge and anxiety symptoms. In unadjusted analyses, higher parental knowledge was marginally associated with lower anxiety symptoms (Table 3). However, after adjusting for parental warmth, the coefficient for parental knowledge changed direction (b = 0.02 in Model 3 and b = 0.01 in Model 4), though it remained non-significant. This reversal may suggest that parental knowledge, when not accompanied by warmth, may not be beneficial or even counterproductive for anxiety symptoms. For example, high parental knowledge in the absence of warmth might reflect intrusive or controlling parenting behaviors, which could exacerbate anxiety in some adolescents. This interpretation aligns with research indicating that monitoring without emotional support can be perceived as overly restrictive or distrustful, particularly in late adolescence [11].

Our findings indicate that immigration background was also associated with depression symptoms in young adulthood. This aligns with a growing body of literature highlighting the mental health burdens faced by immigrant youth, including acculturative stress, discrimination, and socioeconomic disadvantage [39, 40]. While we did not find any significant interactions between parenting practices and immigration background, it is still possible that parenting may operate differently across cultural or migration contexts. For instance, parental warmth might buffer the effects of acculturative stress in some groups, while in others, the challenges of migration and integration may limit the protective role of parenting. Future research should explore these potential moderating effects with larger, more diverse samples and consider qualitative approaches to unpack the mechanisms through which immigration-related stressors influence mental health.

A key strength of this study is the use of a large national cohort of young people in Sweden. Its prospective design, with parenting practices assessed in late adolescence before the measurement of depression and anxiety symptoms in young adulthood, reduces the risk of reverse causation. Furthermore, adjustment for relevant sociodemographic characteristics strengthens the findings by accounting for potential confounding. Yet, the study also presents several limitations. Despite a relatively high response rate, the baseline survey experienced attrition at both the school- and student levels. Although no significant differences in school-level characteristics were observed between participating and non-participating schools [29], student-level attrition across survey waves could indicate potential bias. Student-level attrition may have been systematic, as the survey was conducted during school hours, meaning students absent on the survey day were excluded. Such students may have had, on average, poorer health. Additionally, higher dropout rates were observed among males, individuals whose parents did not have tertiary education, and those with two parents born outside Sweden [41]. These groups may face distinct social and structural challenges such as language barriers, cultural adjustment, or socioeconomic factors that could influence mental health [39, 40] and the ways in which parenting support is accessed or experienced [42, 43]. The exclusion of these students could therefore lead to an underestimation of the true associations between parenting practices and mental health outcomes. This attrition may also have weakened the generalizability of the findings.

Additionally, the use of self-reports for both the predictor and outcome variables may lead to common method variance, potentially exaggerating the observed associations and complicating the interpretation of the results. Further, each parenting measure consisted of only two items, which may limit the depth and reliability of the findings, potentially overlooking important nuances in parenting behaviors. Another limitation concerns the specificity of the parental knowledge measure discussed above. Moreover, the lack of information on depression and anxiety symptoms prior to the third wave, when participants were 21 years old, could also have introduced unmeasured confounding. The reliance on proxy variables to account for mental health at age 18 may not fully capture underlying mental health issues during adolescence, potentially influencing the study’s conclusions, due to the bidirectional nature of associations between parenting factors and depression among adolescents [8]. After all, certain parenting behaviors may emerge as responses rather than antecedents to adolescent depression symptoms. Similarly, Needham et al. [44] demonstrated that parental support and symptoms of depression interact across the transition from adolescence into young adulthood. This underscores the need for more longitudinal studies to capture their complexity over time.

As highlighted in the introduction, parental knowledge is the result of various aspects related to the parent-adolescent relationship, including parental control, solicitation, and adolescent disclosure, yet the measure of knowledge used in the present study does not clarify the source of information held by parents. Access to explicit information on whether parental knowledge stems from adolescent disclosure or parental monitoring efforts may have clarified how these processes differently relate to mental health outcomes. Additionally, the measures of the predictive variables, parental warmth and knowledge, do not differentiate between the parenting practices of mothers and fathers, which may vary and subsequently have different implications for young people’s mental health outcomes [22, 38]. Such analyses may also reveal potential existing gender differences in the relationship between parenting practices and mental well-being [45].

Finally, the observational design of this study limits the ability to draw causal inferences about the relationship between parenting practices and mental health outcomes. While the prospective assessment of parenting practices before mental health outcomes reduces the risk of reverse causation, as noted above, the reliance on proxy measures for baseline mental health may not fully capture potential pre-existing symptoms of depression and anxiety. It is possible that adolescents with poorer mental health prompt different parenting responses, such as increased warmth or monitoring. Thus, the associations observed in this study should be interpreted as correlational rather than causal.

Future research could benefit from qualitative studies that delve into adolescents’ and parents’ experiences with parenting practices, providing a deeper understanding of how parental warmth enhances emotional well-being in adolescence and young adulthood. Such studies may also reveal contextual factors, such as cultural influences and social support networks, that shape these relationships. Furthermore, exploring if there are sensitive periods during adolescence where practices like parental warmth have a heightened impact on later mental health could be valuable for identifying windows of influence. For quantitative research, sourcing mental health outcomes in young adulthood from register data could also be valuable. Although this study did not find any links between parental knowledge and depression or anxiety symptoms when simultaneously accounting for parental warmth, further research could examine the implications of other elements related to monitoring for mental health. Studies using expanded scales, such as those suggested by Stattin and Kerr [9] or more comprehensive monitoring tools [46], could provide richer insights into how different aspects of parental involvement relate to mental health outcomes.

Lastly, research in Sweden and internationally could consider potential cultural differences regarding how parental knowledge is attained and the subsequent implications for mental health. As discussed by Liu et al. [17], behavioral control, defined by setting rules and restrictions, can challenge Western adolescents’ sense of autonomy and self-identity. In contrast, adolescents in cultures where collectivist values emphasize group harmony over individualism, may experience less tension with this type of parental monitoring [17], with different implications for mental health.

Given the high prevalence of internalizing symptoms among adolescents and young adults in countries like Sweden [5], addressing modifiable factors such as parenting offers a possible path for prevention. While evidence-based parenting programs may improve parenting practices [47, 48], broader family and social policies are also likely to influence parenting [49]. Thus, investments in family-centered policies – such as parental leave, work-life balance initiatives, and accessible family support services – may help equip parents to foster warm, supportive relationships with their children, which, in turn, can enhance mental health.

## Conclusion

In conclusion, this study tested the independent effects of parental warmth and knowledge on depression and anxiety symptoms in young adulthood. The findings supported the hypothesis that parental warmth is a protective factor for mental health, while providing no evidence for an independent effect of parental knowledge. These results underscore the importance of fostering warm, supportive parent-adolescent relationships during late adolescence, as these relationships appear to have lasting benefits into young adulthood.

## Supplementary Information

Supplementary Material 1.

Supplementary Material 2.

## Data Availability

The data used for the current study are available from Karolinska Institutet but restrictions apply to the availability of these data, and are therefore not publicly available. Data are however available from the Principal Investigator Dr. Jonas Raninen (jonas.raninen@ki.se) upon reasonable request and with permission of Karolinska Institutet and ethical approval from the Swedish Ethical Review Authority.

## Abbreviations

CI: Confidence Interval
GAD-2: Generalized Anxiety Disorder-2
LLMs: Large language models
LPM: Linear probability models
PHQ-2: Patient Health Questionnaire-2
PHQ-4: Patient Health Questionnaire-4
VIF: Variance inflation factors
