# Supplementary material for: Prospective associations between parental warmth and knowledge in late adolescence and depression and anxiety symptoms in young adulthood: a Swedish cohort study
Source: BMC Public Health. 2026 May 14;26:1560. doi: 10.1186/s12889-026-27712-7 (PMC13173933; doi:10.1186/s12889-026-27712-7)
Supplement: Supplementary file 1 — Supplementary Material 1. [file 12889_2026_27712_MOESM1_ESM.docx]

**Supplementary Material**

**Table S1.** Distributions of variables in the study sample (n=2,697) and among individuals participating in all three waves (n=2,956).

|  | Study sample  (n=2,697) | | Participants in  all three waves  (n=2,956) | |
| --- | --- | --- | --- | --- |
|  | n | % | n | % |
| **Individual and parental background variables** |  |  |  |  |
| Sex |  |  |  |  |
| Males | 1,132 | 42.0 | 1,247 | 42.2 |
| Females | 1,565 | 58.0 | 1,709 | 57.8 |
| *Missing* | - | - | 0 | 0.0 |
| Parental education |  |  |  |  |
| Tertiary | 1,802 | 66.8 | 1,932 | 65.4 |
| ≥3 years secondary | 509 | 18.9 | 547 | 18.5 |
| ≤2 years secondary | 386 | 14.3 | 432 | 14.6 |
| *Missing* | - | - | 45 | 1.5 |
| Immigration background |  |  |  |  |
| Born in Sweden, at least one parent born in Sweden | 2,257 | 83.7 | 2,417 | 81.8 |
| Born in Sweden, both parents born abroad | 233 | 8.6 | 258 | 8.7 |
| Born abroad | 207 | 7.7 | 281 | 9.5 |
| *Missing* | - | - | 0 | 0.0 |
|  |  |  |  |  |
| **Age 18** |  |  |  |  |
|  | n | % | n | % |
| Living arrangements |  |  |  |  |
| Two original parents | 1,744 | 64.7 | 1,868 | 63.2 |
| Single parent | 404 | 15.0 | 440 | 14.9 |
| Shared residence | 305 | 11.3 | 328 | 11.1 |
| Lives in own accommodation | 60 | 2.2 | 71 | 2.4 |
| Other/missing | 184 | 6.8 | 249 | 8.4 |
| *Missing* | - | - | 0 | 0.0 |
| Medication depression | 123 | 4.6 | 143 | 4.8 |
| *Missing* | - | - | 76 | 2.6 |
| Medication anxiety | 136 | 5.0 | 153 | 5.2 |
| *Missing* | - | - | 83 | 2.8 |
|  |  |  |  |  |
|  | Mean | s.d. | Mean | s.d. |
| Parental warmth (n=2,890) | 4.3 | 1.0 | 4.3 | 1.0 |
| Parental knowledge (n=2,883) | 4.5 | 0.8 | 4.4 | 0.9 |
| Psychosomatic complaints (n=2,927) | 7.2 | 2.7 | 7.2 | 2.7 |
|  |  |  |  |  |
| **Age 21** |  |  |  |  |
|  | n | % | n | % |
| Depressive symptoms | 678 | 25.1 | 757 | 25.6 |
| *Missing* | - | - | 45 | 1.5 |
| Anxiety symptoms | 724 | 26.8 | 793 | 26.8 |
| *Missing* | - | - | 43 | 1.5 |

**Table S2.** Pairwise correlations between continuous variables (n=2,697).

|  | Parental warmth  (age 18) | Parental knowledge (age 18) | Psychosomatic complaints (age 18) | Depressive symptoms  (age 21) |
| --- | --- | --- | --- | --- |
|  |  |  |  |  |
| Parental warmth (age 18) | - |  |  |  |
| Parental knowledge (age 18) | 0.38*** | - |  |  |
| Psychosomatic complaints (age 18) | -0.19*** | -0.08*** | - |  |
| Depressive symptoms (age 21) | -0.19*** | -0.10*** | 0.27*** | - |
| Anxiety symptoms (age 21) | -0.16*** | -0.04* | 0.33*** | 0.58*** |

***p < 0.001 *p < 0.05

**Table S3.** Results from linear probability models of depressive symptoms (continuous scale, range 0–6) at age 21, by parenting practices at age 18. Coefficients and 95% confidence intervals (CI) (n=2,697).

|  | Model 1^a^ | | Model 2^b^ | | Model 3^c^ | | Model 4^d^ | |
| --- | --- | --- | --- | --- | --- | --- | --- | --- |
|  | b | 95% CI | b | 95% CI | b | 95% CI | b | 95% CI |
| Parental warmth (age 18) | -0.31*** | -0.38, -0.24 |  |  | -0.29*** | -0.36, -0.21 | -0.20*** | -0.27, -0.13 |
| Parental knowledge (age 18) |  |  | -0.20*** | -0.28, -0.12 | -0.07 | -0.16, 0.01 | -0.06 | -0.14, 0.03 |
| Sex |  |  |  |  |  |  |  |  |
| Males (ref.) |  |  |  |  |  |  | 0.00 |  |
| Females |  |  |  |  |  |  | -0.03 | -0.16, 0.09 |
| Parental education |  |  |  |  |  |  |  |  |
| Tertiary (ref.) |  |  |  |  |  |  | 0.00 |  |
| ≥3 years secondary |  |  |  |  |  |  | -0.02 | -0.17, 0.13 |
| ≤2 years secondary |  |  |  |  |  |  | 0.12 | -0.05, 0.30 |
| Immigration background |  |  |  |  |  |  |  |  |
| Born in Sweden, at least one parent born in Sweden (ref.) |  |  |  |  |  |  | 0.00 |  |
| Born in Sweden, both parents born abroad |  |  |  |  |  |  | 0.30** | 0.08, 0.52 |
| Born abroad |  |  |  |  |  |  | 0.44** | 0.21, 0.67 |
| Living arrangements (age 18) |  |  |  |  |  |  |  |  |
| Two original parents (ref.) |  |  |  |  |  |  | 0.00 |  |
| Single parent |  |  |  |  |  |  | 0.03 | -0.15, 0.20 |
| Shared residence |  |  |  |  |  |  | 0.06 | -0.12, 0.24 |
| Lives in own accommodation |  |  |  |  |  |  | 0.01 | -0.34, 0.37 |
| Other/missing |  |  |  |  |  |  | 0.12 | -0.14, 0.39 |
| Psychosomatic complaints (age 18) |  |  |  |  |  |  | 0.13*** | 0.10, 0.15 |
| Medication depression (age 18) |  |  |  |  |  |  | 0.63** | 0.19, 1.07 |
| Medication anxiety (age 18) |  |  |  |  |  |  | 0.18 | -0.23, 0.60 |
|  |  |  |  |  |  |  |  |  |
| R^2^ | 0.04 |  | 0.01 |  | 0.04 |  | 0.11 |  |

***p < 0.001 **p < 0.01 *p < 0.05

^a^ Includes only parental warmth.

^b^ Includes only parental knowledge.

^c^ Mutually adjusts for parental warmth and parental knowledge.

^c^ Mutually adjusts for parental warmth, parental knowledge, and covariates.

**Table S4.** Results from linear probability models of anxiety symptoms (continuous scale, range 0–6) at age 21, by parenting practices at age 18. Coefficients and 95% confidence intervals (CI) (n=2,697).

|  | Model 1^a^ | | Model 2^b^ | | Model 3^c^ | | Model 4^d^ | |
| --- | --- | --- | --- | --- | --- | --- | --- | --- |
|  | b | 95% CI | b | 95% CI | b | 95% CI | b | 95% CI |
| Parental warmth (age 18) | -0.30*** | -0.37, -0.22 |  |  | -0.32*** | -0.40, -0.23 | -0.21*** | -0.29, -0.13 |
| Parental knowledge (age 18) |  |  | -0.08* | -0.16, 0.00 | 0.06 | -0.03, 0.15 | 0.02 | -0.07, 0.10 |
| Sex |  |  |  |  |  |  |  |  |
| Males (ref.) |  |  |  |  |  |  | 0.00 |  |
| Females |  |  |  |  |  |  | 0.62*** | 0.48, 0.75 |
| Parental education |  |  |  |  |  |  |  |  |
| Tertiary (ref.) |  |  |  |  |  |  | 0.00 |  |
| ≥3 years secondary |  |  |  |  |  |  | -0.14 | -0.30, 0.02 |
| ≤2 years secondary |  |  |  |  |  |  | -0.09 | -0.26, 0.08 |
| Immigration background |  |  |  |  |  |  |  |  |
| Born in Sweden, at least one parent born in Sweden (ref.) |  |  |  |  |  |  | 0.00 |  |
| Born in Sweden, both parents born abroad |  |  |  |  |  |  | 0.10 | -0.12, 0.33 |
| Born abroad |  |  |  |  |  |  | 0.16 | -0.11, 0.44 |
| Living arrangements (age 18) |  |  |  |  |  |  |  |  |
| Two original parents (ref.) |  |  |  |  |  |  | 0.00 |  |
| Single parent |  |  |  |  |  |  | 0.11 | -0.07, 0.30 |
| Shared residence |  |  |  |  |  |  | 0.15 | -0.04, 0.35 |
| Lives in own accommodation |  |  |  |  |  |  | -0.08 | -0.49, 0.32 |
| Other/missing |  |  |  |  |  |  | 0.12 | -0.14, 0.39 |
| Psychosomatic complaints (age 18) |  |  |  |  |  |  | 0.16*** | 0.13, 0.18 |
| Medication depression (age 18) |  |  |  |  |  |  | 0.46 | -0.01, 0.92 |
| Medication anxiety (age 18) |  |  |  |  |  |  | 0.33 | -0.12, 0.79 |
|  |  |  |  |  |  |  |  |  |
| R^2^ | 0.03 |  | 0.00 |  | 0.03 |  | 0.16 |  |

***p < 0.001 **p < 0.01 *p < 0.05

^a^ Includes only parental warmth.

^b^ Includes only parental knowledge.

^c^ Mutually adjusts for parental warmth and parental knowledge.

^c^ Mutually adjusts for parental warmth, parental knowledge, and covariates.
